# Acute Respiratory Distress Syndrome Caused by Human Adenovirus in Adults: A Prospective Observational Study in Guangdong, China

**DOI:** 10.3389/fmed.2021.791163

**Published:** 2022-01-27

**Authors:** Zhan Wu, Rong Zhang, Dongdong Liu, Xuesong Liu, Jierong Zhang, Zhihui Zhang, Sibei Chen, Weiqun He, Yimin Li, Yonghao Xu, Xiaoqing Liu

**Affiliations:** Department of Critical Care Medicine, State Key Laboratory of Respiratory Diseases, National Clinical Research Center for Respiratory Disease, Guangzhou Institute of Respiratory Health, The First Affiliated Hospital of Guangzhou Medical University, Guangzhou, China

**Keywords:** acute respiratory distress syndrome, adenovirus, severe pneumonia, infection, adults

## Abstract

**Background:**

Viral causes of acute respiratory distress syndrome (ARDS) are mostly limited to influenza. However, adenovirus has been emerging as a cause of ARDS with a high mortality rate and described in adults are rare.

**Methods:**

We conducted a prospective, single-center observational study of viral pneumonia with ARDS and confirmed adenovirus-associated ARDS in adults at our quaternary referral institution between March 2019 and June 2020. We prospectively analyzed clinical characteristics, laboratory test results, radiological characteristics, viral load from nasopharyngeal swabs and endotracheal aspirates, treatments, and outcomes for the study participants.

**Results:**

The study enrolled 143 ARDS patients, including 47 patients with viral pneumonia-related ARDS, among which there were 14 adenovirus-associated ARDS patients, which accounted for 29.79% of the viral pneumonia-related ARDS cases. Among the adenovirus-associated ARDS patients, 78.57% were men with a mean age of 54.93 ± 19.04 years, younger than that of the non-adenovirus associated ARDS patients. Adenovirus-associated ARDS patients had no specific clinical characteristics, but they presented with decrease in the number of CD3+CD4+ T cells and higher serum creatinine during the early stage. The viral load and the positivity rate in the lower respiratory tract were higher than that of the upper respiratory tract in the patients with adenovirus-associated ARDS. All patients required invasive mechanical ventilation treatment. The average time from shortness of breath to the application of invasive ventilation was 24 h. Ten patients (71.43%) complicated by acute kidney injury, while 13 patients (71.43%) in the non-adenovirus associated ARDS group (*P* = 0.045). Additionally, 85.71% of the 14 adenovirus-associated ARDS patients survived. No significant differences were detected between the two groups regarding duration of ventilation, length of ICU stay and mortality.

**Conclusion:**

Adenovirus infection is an important cause of virus-related ARDS. The positivity rate of adenovirus infection in lower respiratory tract secretions was higher than that in upper respiratory tract secretions in these patients. Age, lower CD3+CD4+ T cells, and high serum creatinine may be were associated with adenovirus induce ARDS in adults required mechanical ventilation. Early identification and intervention to prevent disease progression are essential for reducing the mortality rate in these patients.

## Introduction

Acute respiratory distress syndrome (ARDS) is a rapid inflammatory lung injury with a high mortality rate that ranges from 30.0-46.0% (1–3). Infectious pneumonia is the most common disease leading to ARDS, with viral infection accounting for ~22-40% of cases (4, 5). Influenza and rhinovirus have been most commonly detected in viral pneumonias followed by parainfluenza, adenovirus, respiratory syncytial virus, coronavirus, and human metapneumovirus (4, 6). Adenovirus have been recently found to be responsible for infection in ventilated or non-ventilated ARDS patients. However, the proportion of viral pneumonia leading to ARDS is unknown and the true impact of these viral infections on outcomes remains to be determined.

Some viral infections, such as influenza A H1N1, H5N1, and H7N9 and the coronaviruses severe acute respiratory syndrome (SARS), severe acute respiratory syndrome coronavirus-2 (SARS-CoV-2), and Middle East Respiratory Syndrome (MERS), are associated with a high incidence of ARDS and increased mortality (7, 8). Adenovirus-associated ARDS has been reported in adult patients with rapid progression to multi-organ failure and death, which has raised concerns (9). However, there is limited information on adult adenovirus-associated ARDS and no consensus on its management (10).

Human adenoviruses (HAdVs) are non-enveloped DNA viruses that are associated with a wide range of clinical manifestations (11). Adenovirus infection that causes severe fatal pneumonia has been well described in infants and children, but reports in adults are rare (12). HAdV-associated ARDS infection is a devastating disease with rapid progression to multi-organ failure and death, and it can be fulminant with a mortality rate of ~40% in patients who require mechanical ventilation (13). However, ARDS caused by HAdV in adults required mechanical ventilation was rarely reported. Here, we performed a prospective, observational study to evaluate the proportion of viral pneumonia leading to ARDS in adult patients, we also analyzed to compare sequential viral load test results on nasopharyngeal swabs and endotracheal aspirates, and finally set a comparison group to reveal the risk factors of adenovirus-associated ARDS requiring mechanical ventilation.

## Methods

### Study Design and Population

We performed a prospective, single-center observational study in adult patients with viral pneumonia with ARDS who were admitted to our respiratory intensive care unit (ICU) between March 2019 and June 2020. The study was approved by the Ethics Committee of the First Affiliated Hospital of Guangzhou Medical University. All patients provided written informed consent for their data to be used for research.

Patients were included if they met the following inclusion criteria: age ≥ 18 years, patients receiving invasive mechanical ventilation with a diagnosis of ARDS <24 h before enrollment in the trial. ARDS was diagnosed in accordance with the Berlin definition (2), as follows: (1) developing within 1 week of a known clinical insult or new or worsening respiratory symptoms; (2) bilateral opacities not fully explained by effusions, lobar and/or lung collapse, or nodules; (3) respiratory failure not fully explained by cardiac failure or fluid overload; (4) partial oxygen pressure/fraction of inspired oxygen (PaO_2_/FiO_2_) ≤300 mmHg with positive end-expiratory pressure (PEEP) ≥5 cmH_2_O; and (5) a chest radiograph with three or four quadrants with opacities. Patients with HAdV or other virus's infection confirmed by chest X-ray, a positive RT-PCR finding from an airway specimen, requiring invasive mechanical ventilation were included in the analysis. The exclusion criteria were age younger than 18 years; pregnant women, or inability or unwillingness to provide informed consent.

### Clinical Data Collection

A standardized electronic case report form was used to prospectively collect the study data. The following data were obtained: age, sex, body mass index (BMI), preexisting comorbidities, Acute Physiology and Chronic Health Evaluation II (APACHEII) score, daily Sequential Organ Failure Assessment (SOFA) score, and clinical pulmonary infection score (CPIS). Clinical symptoms (fever, cough, sputum, and dyspnea), signs (body temperature, heart rate, respiratory frequency, and blood pressure), and laboratory data (procalcitonin, white blood cell count, lymphocyte, platelet count, and creatinine level), microbiological findings, and images of the lung (chest X-ray and computed tomography) were also collected. Additionally, complications, treatments, respiratory support, and clinical outcomes were recorded.

### Molecular Assay for Respiratory Virus Detection

Nasopharyngeal swabs and endotracheal aspirates (ETA) were collected at admission and during hospitalization and stored at −80°C until testing. All nasopharyngeal swabs and ETA samples were analyzed using multiplex quantitative polymerase chain reaction (Q-PCR) technology. Viral nucleic acid was extracted from the samples using the QIAamp MiniElute Virus Spin kit (QIAGEN, Germany) following the manufacturer's instructions. This assay tested for 24 respiratory viruses (adenovirus; influenza virus type A H1N1, H3N2, H1N1 2009, H5N1, and H7N9; influenza virus type B; respiratory syncytial virus types A and B; parainfluenza viruses type 1, 2, 3, and 4; metapneumovirus; coronavirus OC43, 229E, NL63, HKU1, SARS, and MERS; rhinovirus; bocavirus; Nipah virus; and circovirus).

The viral load was indicated as the cycle threshold (Ct) value. Ct values were used to analyze each patient's viral load. Ct values were inversely correlated to the quantity of RNA target present in the specimen. A Ct value of <40 was defined as positive for the viruses and >40 was defined as a negative result. Samples with a Ct value between 37 and 40 were retested at least twice.

### Statistical Analysis

Measurement variables were summarized using the median (interquartile range) or the mean and range, and enumeration variables were presented using the frequency and percentage. Differences between groups were assessed with the Fisher exact test for categorical variables and the Mann-Whitney *U*-test for continuous variables, and *p* < 0.05 was considered to be significant. Statistical analysis was performed using GraphPad Prism 7.0.

## Results

**Figure 1:**
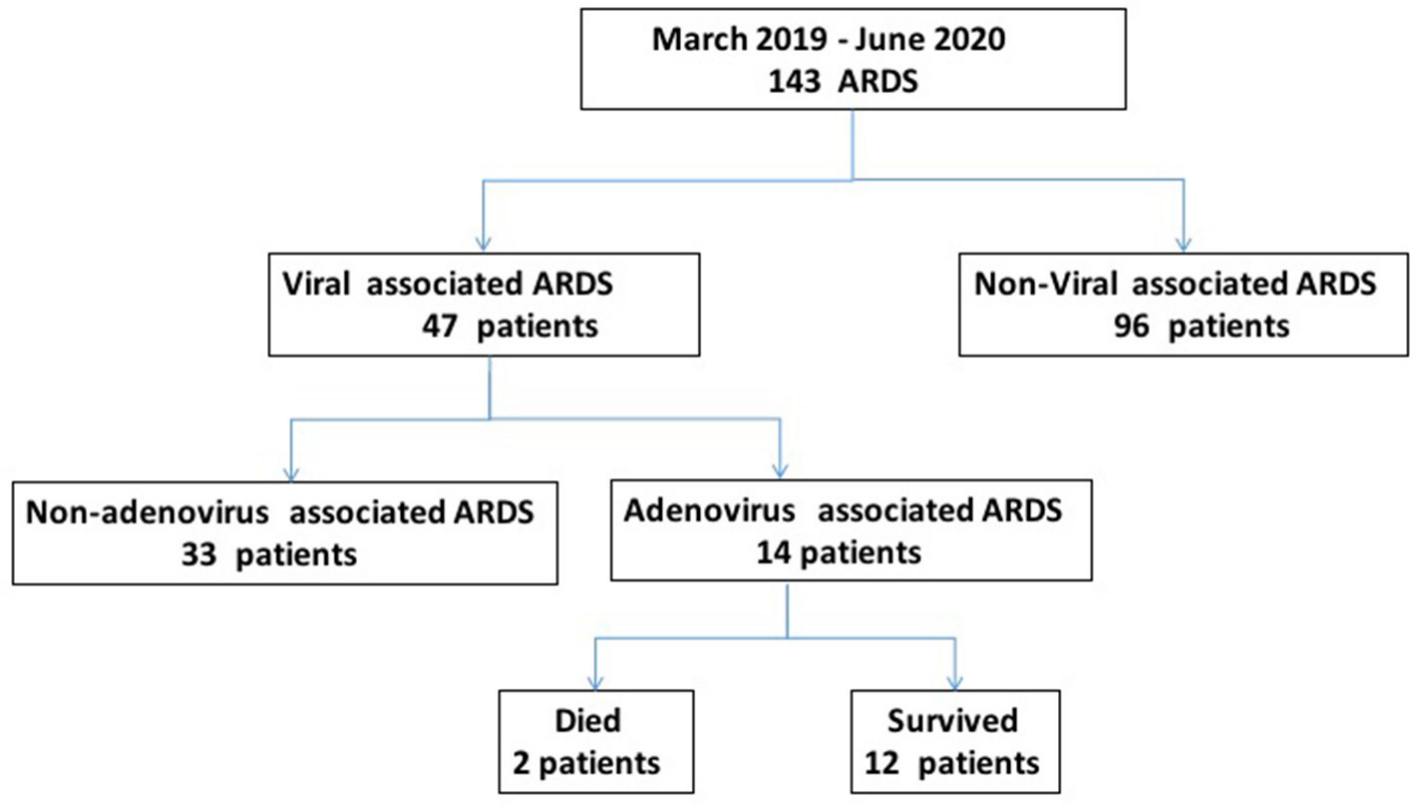
Flowchart of patient groups in the study. ARDS, acute respiratory distress syndrome.

**Figure 2:**
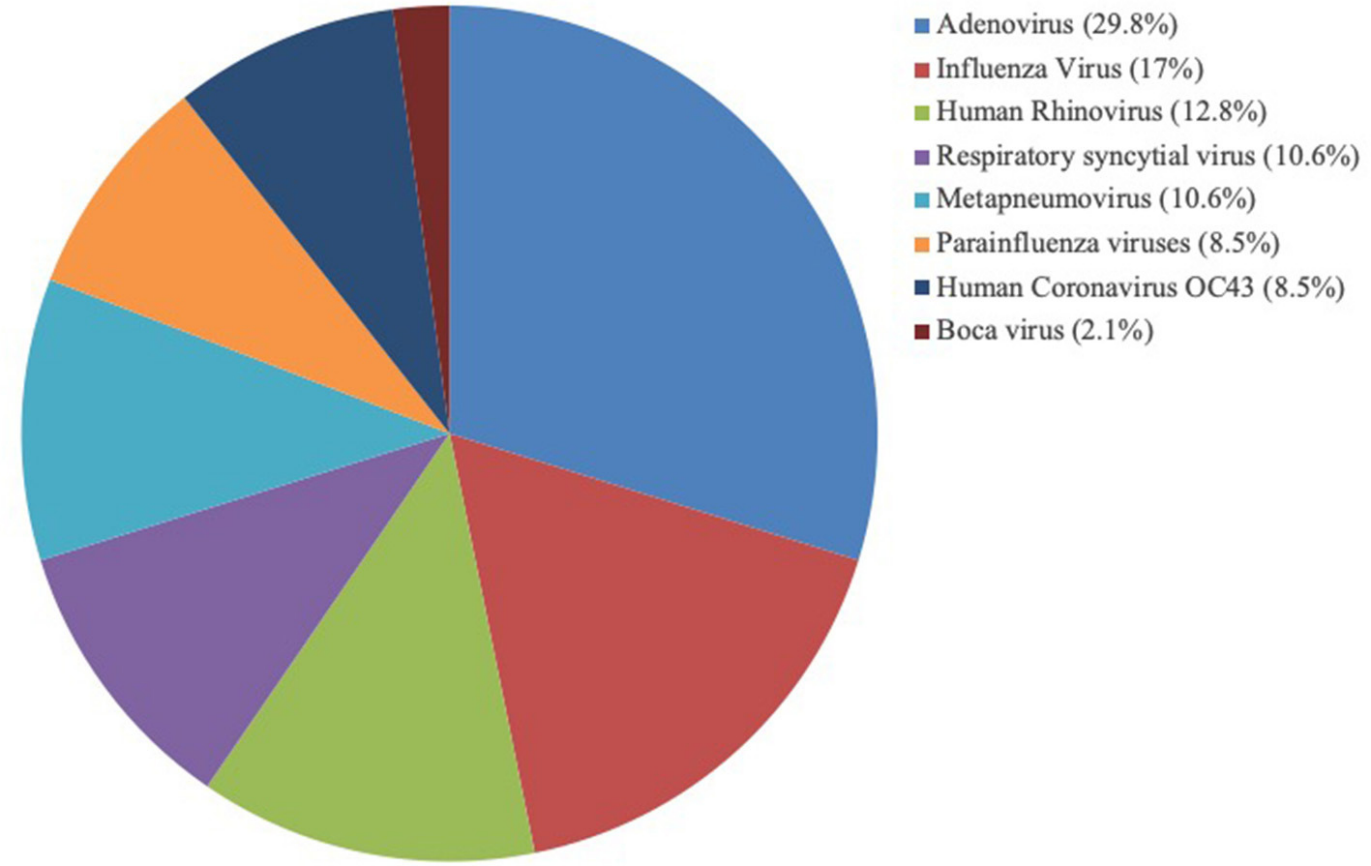
Distribution of viruses detected in 47 ARDS patients.

The study included 143 patients, including 47 patients (32.87%) with viral pneumonia-related ARDS and 14 adenovirus-positive ARDS patients, which accounted for 29.79% of viral pneumonia-related ARDS (Figure 1). As shown in Figure 2, among the non-adenovirus associated ARDS patients, the most common finding was Influenza virus, detected in 17% of the samples, followed by human rhinovirus (12.8%), respiratory syncytial virus (10.6%), and human metapneumovirus (10.6%), parainfluenza viruses (8.5%), human coronavirus OC43 (8.5%), boca virus (2.1%).

**Figure 3:**
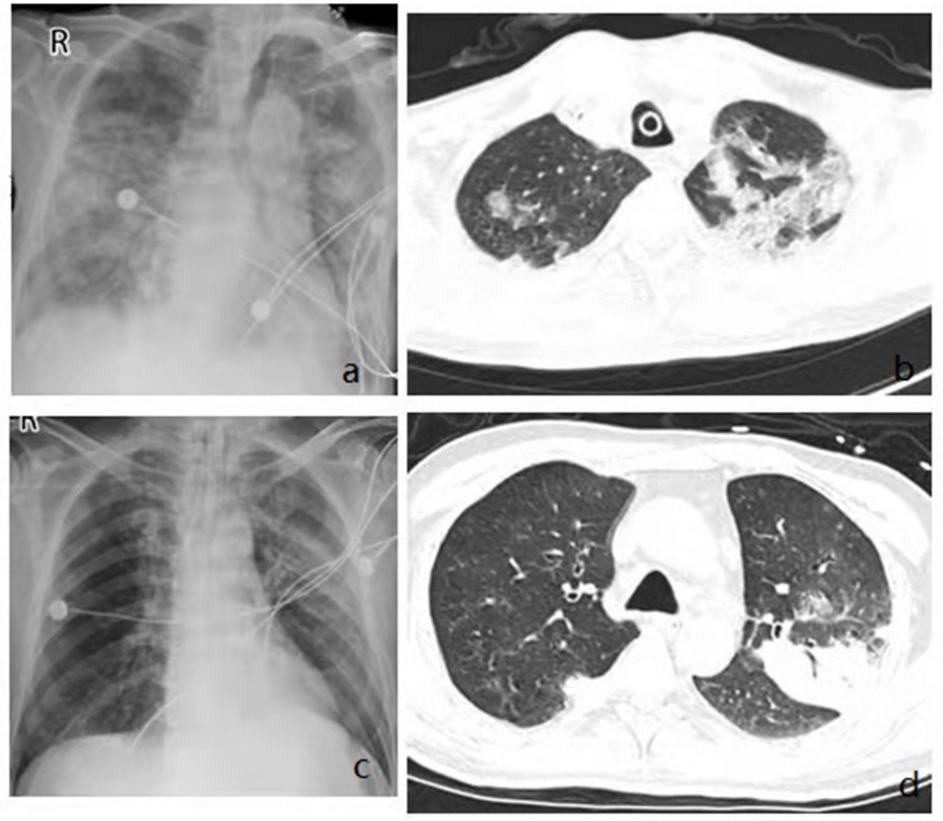
Imaging findings showing diffuse multifocal or diffuse opacity. Chest x rays from admission showing bilateral diffuse infiltrates **(a,c)**, Chest CT from admission showing multifocal consolidation and groundglass opacity in both lungs **(b,d)**.

The general condition and clinical characteristics of the patients with adenovirus-associated ARDS and non-adenovirus associated ARDS are shown in Table 1. The mean age was 54.93 ± 19.04 years among the adenovirus-associated ARDS patients, younger than that of the non-adenovirus associated ARDS patients. Among the adenovirus-associated ARDS patients, 78.57% were males, 50% were smokers, and 71.43% had high nutritional risk (Table 1). In addition, 28.57% of the adenovirus-associated patients had concurrent lung diseases, and 28.57% of the patients also had an immunodeficiency. All patients had cough, sputum expectoration, and shortness of breath, and 92.86% of them had a fever. Most patients presented with a low-grade fever. As shown in Table 2, the PaO_2_/FiO_2_ for 64.29% of the adenovirus-associated patients was between 150 and 200 mmHg. Most of the patients had normal blood CO_2_, manifesting as type I respiratory failure. The average time from the onset of shortness of breath to tracheal intubation was 24 h. Hypoproteinemia occurred in 85.71% of the adenovirus-associated ARDS patients, and abnormal coagulation function occurred in >60% of the patients. At the time of enrollment, 64.29% of the patients had a low total number of white blood cells, and more than 70% of the patients had a decrease in the total number and percentage of lymphocytes. Additionally, 85.71% of the adenovirus-associated patients had a significant decrease in the number of CD3+CD4+ T cells and higher serum creatinine during the early stage compared with the non-adenovirus associated ARDS patients. Most of the adenovirus-associated patients had diffuse infiltrates in both lungs. Three (21.43%) of the patients had interstitial abnormalities. Five patients (35.71%) had pleural effusions. Only a few patients showed ground-glass opacity (Figure 3).

**Table 1:** Demographic data and clinical characteristics of the patients with adenovirus-associated ARDS and non-adenovirus associated ARDS.

	**Adenovirus associated ARDS (*n* = 14)**	**Non-adenovirus associated ARDS (*n* = 33)**	***P*-value**
Sex(male)	11(78.57%)	23(69.70%)	0.79
Age(years)	54.93 ± 19.04	64.40 ± 11.20	0.05
BMI (kg/m^2^)	20.30 ± 3.03	20.76 ± 6.83	0.81
APACHEII score	19.14 ± 7.35	21.06 ± 8.37	0.46
SOFA score	10.50 ± 4.09	8.94 ± 3.77	0.21
CPIS score	6.50 ± 1.15	6.06 ± 0.93	0.60
Smoking	7(50%)	11(33.33%)	0.28
High nutritional risk	9(64.29%)	25(75.76%)	0.65
Underlying lung disease	4(28.57%)	12(36.36%)	0.86
COPD	2(14.28%)	11(33.33%)	0.33
Bronchiectasis	1(7.14%)	1(3.03%)	0.52
Interstitial lung disease	1(7.14%)	1(3.03%)	0.52
Immunodeficiency	4(28.57%)	8(24.24%)	>0.99
**Symptoms**			
Fever	13(92.86%)	23(69.70%)	0.18
Cough	14(100.00%)	33(100%)	
Shortness of breath	14(100.00%)	33(100%)	
**Temperature (** **°** **C)**			
36.0-37.2	7(50%)	23(69.70%)	0.20
37.3-38.0	5(35.71%)	4(12.12%)	0.14
38.1-39.0	1(7.14%)	5(15.15%)	0.45
39.1-41	1(7.14%)	1(3.03%)	0.52
Heart rate(bpm)	98.36 ± 24.11	101.58 ± 26.00	0.70
Respiratory rate(bpm)	22.93 ± 6.96	23.36 ± 7.23	0.85
MAP (mmHg)	75.86 ± 12.27	77.86 ± 10.35	0.74
**Co-infection**			
Other viruses	4(28.57%)	0	
Influenza virus	2(14.29%)	0	
Human Coronavirus OC43	1(7.14%)	0	
Human rhinovirus	1(7.14%)	0	
Bacteria	10(71.43%)	23(69.70%)	>0.99
*Acinetobacter baumannii*	6(42.86%)	8(24.24%)	0.35
*Stenotrophomonas Maltophilia*	3(21.43%)	7(21.21%)	>0.99
*Klebsiella pneunoniae*	2(14.29%)	3(9.09%)	>0.99
Other bacteria	4(28.57%)	7(21.21%)	0.87

**Table 2:** Laboratory findings and radiographic results for patients with adenovirus-associated ARDS.

**Characteristic**	**Adenovirus associated ARDS (*n* = 14)**	**Non-adenovirus associated ARDS (*n* = 33)**	***P*-value**
White blood cell count, × 10^9^/L	12.66 ± 6.40	11.95 ± 6.67	0.84
<4	0	3(9.09%)	0.24
>10	9(64.29%)	14(42.42%)	0.17
Platelet count, × 10^9^/L	165.43 ± 103.26	168.24 ± 75.45	0.92
<100	5(35.71%)	6(18.18%)	0.36
Lymphocyte, × 10^9^/L	0.64 ± 0.49	0.54 ± 0.24	0.59
<0.9	10(71.43%)	28(84.80%)	0.51
CD3+CD45+ T, Cell/Ul	278.00(171.50-634.00)	343.00(138.00-665.00)	0.67
<955	12(85.71%)	24(72.73%)	0.56
CD3+CD4+ T, Cell/Ul	148.00(106.00-365.00)	237.00(101.00-596.00)	0.08
<550	12(85.71%)	18(54.55%)	0.041
CD3+CD8+ T, Cell/Ul	120.00(57.50-282.00)	107.00(62.00-259.00)	0.91
<320	11(78.57%)	22(66.66%)	0.64
Interleukin-6, pg/ml	39.37(12.53-143.06)	22.93(12.40-140.14)	0.84
>5.3	12(85.71%)	22(66.66%)	0.33
Procalcitonin, ng/ml	0.38(1.90-4.66)	0.29(1.69-5.43)	0.98
**Arterial blood gas analysis:**			
pH	7.37 ± 0.09	7.37 ± 0.12	0.90
pCO_2_, mmHg	44.12 ± 6.83	47.09 ± 10.26	0.33
P/F	168.59 ± 41.77	153.13 ± 53.77	0.34
<150	5(35.71%)	20(60.61.%)	0.12
150-200	9(64.29%)	8(24.24%)	0.01
Albumin, g/L	30.96 ± 10.14	31.21 ± 6.89	0.92
<35	12(85.71%)	28(84.85%)	>0.99
Prothrombin time, s	16.06 ± 2.73	16.24 ± 2.15	0.80
>14.5	10(71.43%)	29(87.88.%)	0.34
Activated partial thromboplastin time, s	51.23 ± 28.21	48.98 ± 20.04	0.76
>42.8	9(64.29%)	16(48.48%)	0.32
Creatinine, umol/L	162.55(87.50-282.50)	73.0(60.0-117.30)	0.047
>133	9(64.29%)	7(21.21%)	0.01
**Abnormalities on chest radiograph**			
Ground-glass opacity	2(14.29%)	1(3.03%)	0.43
Bilateral patchy shadowing	14(100%)	29(87.88%)	0.17
Interstitial abnormalities	3(21.43%)	4(12.12%)	0.71
Pleural effusions	5(35.71%)	6(18.18%)	0.36

**Figure 4:**
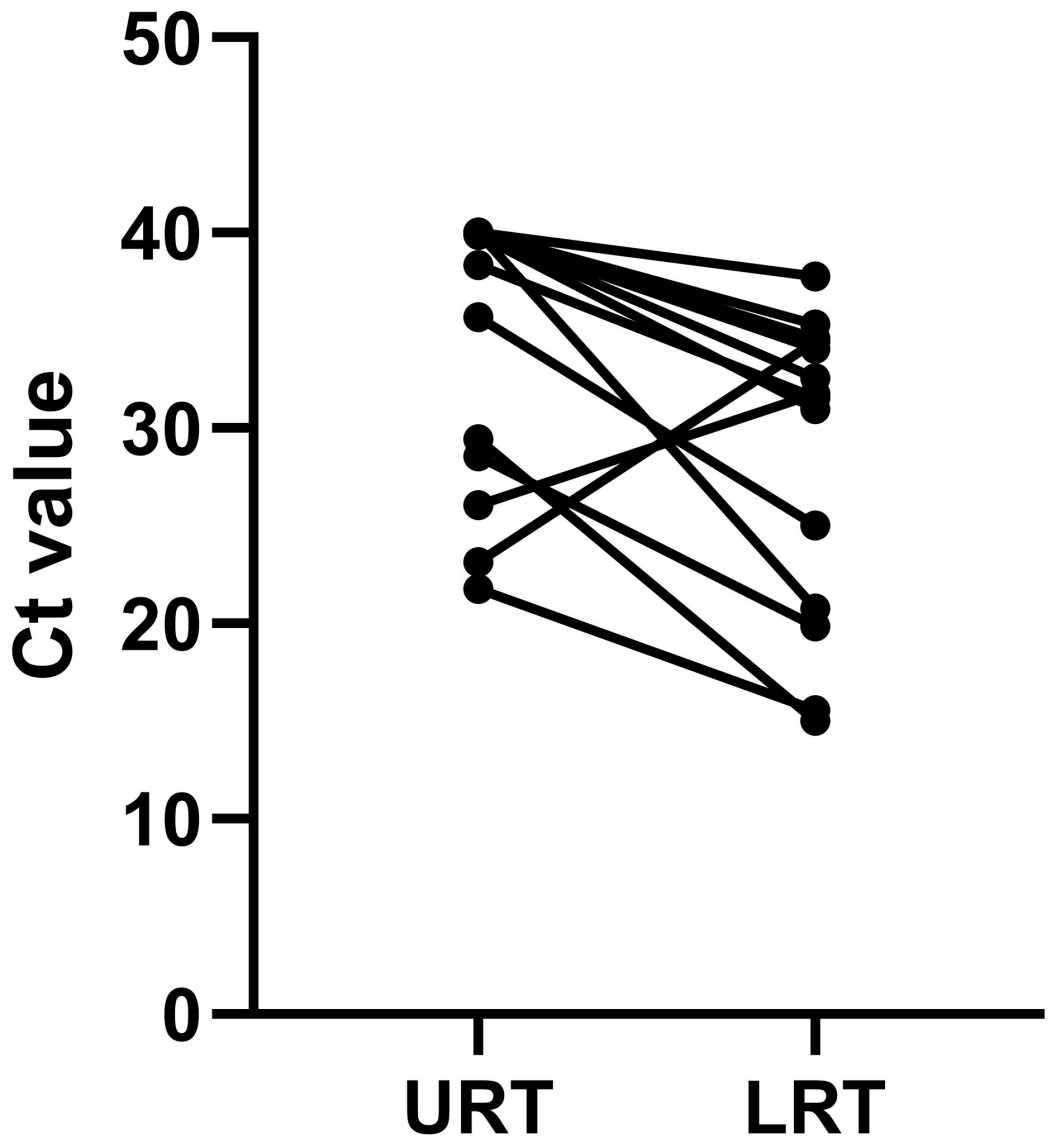
Comparison of the Ct value between URT and LRT samples upon admission to the ICU. URT, upper respiratory tract; LRT, lower respiratory tract; ICU, intensive care unit; Ct, cycle threshold.

**Figure 5:**
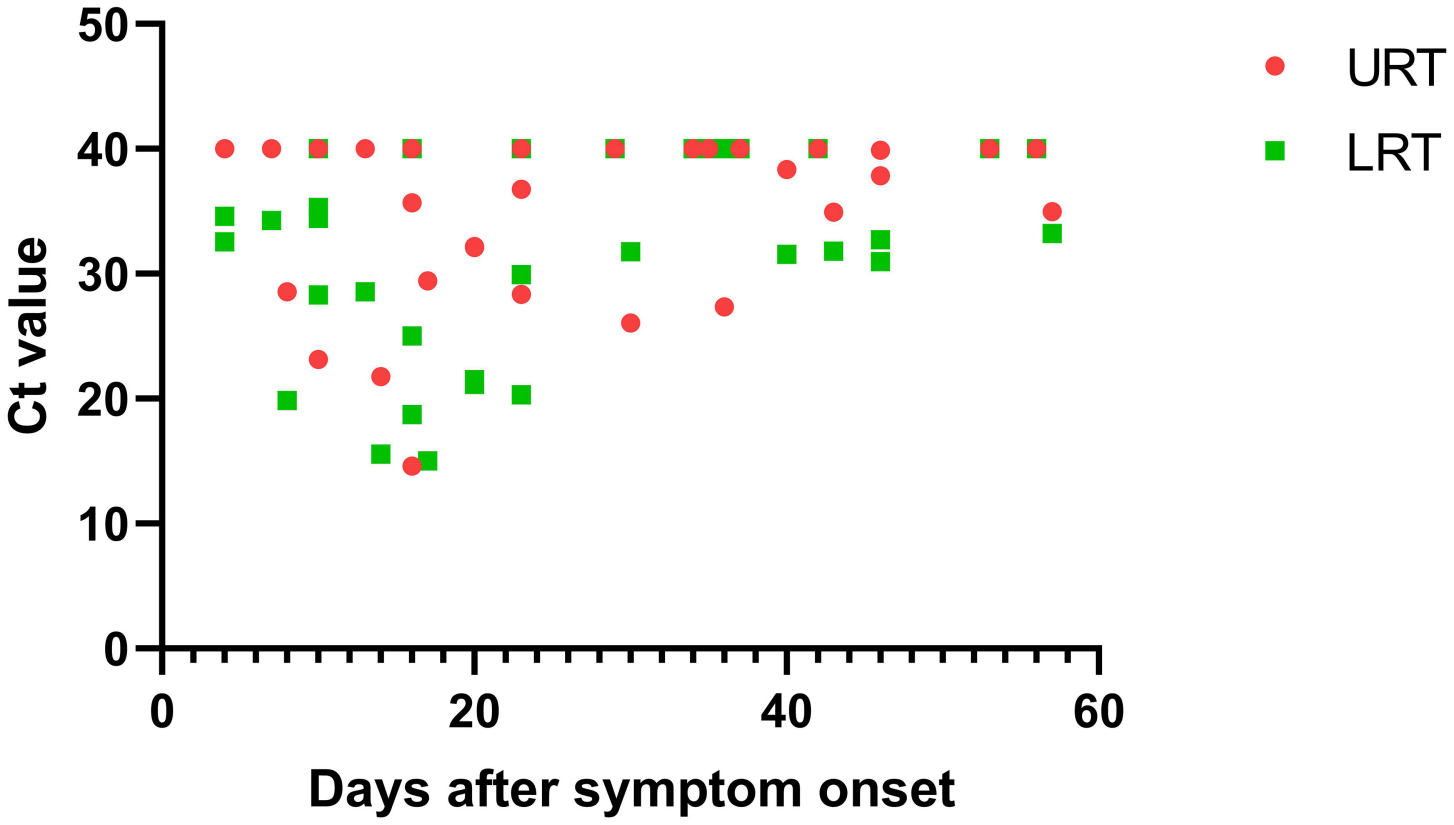
The relationship between Ct value and days after symptom onset in the URT and LRT samples. URT, upper respiratory tract; LRT, lower respiratory tract; ICU, intensive care unit; Ct, cycle threshold.

Within 48 h of enrollment, paired upper respiratory tract (URT) samples (nasopharyngeal swab) and lower respiratory tract (LRT) samples (sputum and bronchoalveolar lavage) were collected from the adenovirus-associated ARDS patients to detect the virus using multiplex Q-PCR. As shown in Table 3, the LRT samples showed a significantly higher positive rate (100%, 14/14) than URT samples (64.29%, 9/14) for detecting respiratory viruses (*p* < 0.001), and LRT samples had higher levels of detection using a Ct value of 31.66 (20.56–34.78) compared with URT sampling using a Ct value of 39.14 (27.95–40) (Figure 4). On average, URT sites cleared faster than LRT sites (Figure 5). Detection of other viruses, fungi, and bacteria from the sputum collected from patients within 48 h of enrollment showed that four patients (28.57%) also had another viral infection, and among these patients, the most common virus was the influenza virus that accounted for 14.29% of viral infections. Nine patients (64.29%) had positive bacterial cultures in the sputum, with the highest positivity rate for Acinetobacter baumannii, which was detected in six patients (42.86%). All patients had a negative fungal culture from the sputum, blood, and bronchoalveolar lavage fluid in (1-3)-β-D-glucan (G) and galactomannan (GM) tests.

**Table 3:** Comparison of multiplex Q-PCR results between URT and LRT samples.

	**URT**	**LRT**	***P*-value**
Detected *n* (%)	9(64.29%)	14(100%)	<0.001
Undetected *n* (%)	5(35.71%)	0	<0.001
Time of virus clearance(days)	15.16(4-37)	24.17(6-42)	<0.001

All patients received antiviral therapy, including ganciclovir (5 mg/kg intravenously every 12 h) and oseltamivir. Considering that bacterial coinfection may combine with a severe viral infection, broad-spectrum intravenous antibiotics were administered to all patients. Additionally, 78.57% of the patients received immunoglobulin. All patients required invasive ventilation, the median duration of invasive mechanical ventilation was 22 days (14–75 days), compared with 22 days (range, 10–34 days) in the non-adenovirus associated ARDS group. Ten patients (71.43%) complicated by acute kidney injury, while 13 patients (71.43%) in the non-adenovirus associated ARDS group (*P* = 0.045). Three patients (21.43%) required extracorporeal membrane oxygenation (ECMO), with a treatment duration of 7 to 12 days, and one patient (7.14%) underwent prone position ventilation. The average length of ICU stay was 26.50 days (IQR, 15–75 days), and the median length of hospital stay was 37.5 days (IQR, 29.75–81.00 days). Among the 14 patients in this study, there were two deaths, with a mortality rate of 14.29% during the study period (Table 4). No significant differences were detected between the two groups regarding with the mortality.

**Table 4:** Complications, treatments, and clinical outcomes in patients with adenovirus-associated ARDS.

**Characteristic**	**Adenovirus associated ARDS (*n* = 14)**	**Non-adenovirus associated ARDS (*n* = 33)**	***P-*value**
**Complications**			
AKI	10(71.43%)	13(39.39%)	0.045
Sepsis shock	4(28.57%)	12(36.36%)	0.86
Pneumothorax	2(14.29%)	0	0.02
**Treatments**			
CRRT	6(42.86%)	10(30.30%)	0.62
ECMO	3(21.43%)	2(6.06%)	0.30
Prone position ventilation	1(7.14%)	2(6.06%)	0.89
Vasoactive	11(78.57%)	25(75.76%)	>0.99
Muscle relaxants	11(78.57%)	20(60.61%)	0.39
Intravenous immune globulin	6(42.86%)	10(30.30%)	0.62
**Anti-viral agents**			
Oseltamivir	7(50.00%)	11(33.33%)	0.28
Ganciclovir	11(78.57%)	9(27.27%)	0.001
**Clinical outcomes**			
Duration of dyspnea to IMV	1.00(1.00-2.00)	3.00(2.00-5.00)	>0.99
Duration of IMV (days)	22.00(14.00-75.25)	22.00(10.00-34.00)	0.41
Length of ICU stay(days)	26.50(15.75-75.25)	27.00(11.50-41.50)	0.52
Length of hospital stay(days)	37.50(29.75-81.00)	46.00(27.50-57.50)	0.89
Death	2(14.29%)	4(12.12%)	>0.99

## Discussion

The number of ARDS cases caused by viral pneumonia is increasing, resulting in a high mortality rate (14). Previous studies have mostly focused on influenza viruses, such as H1N1 and H7N9 (15, 16). Cases of adenovirus-associated ARDS have gradually been increasing, which may be due to recent developments in molecular diagnostic technology (17). However, there is limited data about the viral etiology of ARDS in patients who required mechanical ventilation. Zhou et al. reported that the incidence of adenovirus pneumonia ranks third among viral pneumonia in adults in China (6). They have also found that among virus-related ARDS patients with PO_2_/FiO_2_ ratio <200 mmHg, HAdV infection was the most frequently detected virus (6). Our study showed that in all ARDS patients, virus-related ARDS accounted for 32.87% of infections. Among these infections, adenovirus-associated ARDS accounted for 9.79% of all ARDS patients and 29.79% of virus-related ARDS patients. The prevalence of respiratory viruses varies in different countries and different populations (4–6, 13). To the best of our knowledge, this is the first study about the viral etiology in ARDS patients who required mechanical ventilation in China.

There are few studies on adenovirus-associated ARDS in adults who required mechanical ventilation (18–20). In 2000, two non-immunocompromised soldiers became infected with adenovirus, which resulted in ARDS (21). In 2006, there was an outbreak of adenovirus pneumonia caused by HAdV-B11 in the USA; 140 people were diagnosed with HAdV infection, and 24 patients who were diagnosed with ARDS were admitted to the ICU (22). Some studies have proposed that severe adenovirus infection is likely to occur in children and immunocompromised adults, such as HIV patients, and patients after transplantation (23, 24). In this study, most of the adenovirus-associated ARDS patients, with a mean age of 54 years, had no underlying diseases. Among these patients, 78.57% of them were men, and 50% of the patients were smokers, suggesting that severe adenovirus pneumonia in non-immunocompromised adults was likely to occur in middle-aged men. Additionally, 85.71% of the patients had a significant decrease in the number of CD3+CD4+ T cells during the early stage, which suggests that adenovirus infection may cause immune system dysregulation.

Delayed clearance of respiratory adenovirus infection leads to a worse prognosis in these patients, and monitoring the viral load may help to predict the disease severity and the patients' prognosis (25, 26). Rapid identification of adenovirus viral infection is critical to reduce the overall costs of patient management. Multiplex Q-PCR is of great value in the early diagnosis of virus infection because of its high sensitivity (27, 28). However, viral testing of URT and LRT samples may yield different results (29). Currently, few studies have been published that compare the diagnostic yields of URT and LRT samples to detect adenovirus. In our study, the detection rates of adenovirus from LRT and URT samples were 100 and 64.29%, respectively. Similarly, a European study reported that the overall virus positivity rate of URT was lower than that of the LRT specimens (24.5 vs. 44.2%) (30). In this study, the percentage of positive specimens was higher in LRT than in URT specimens. On average, URT specimens cleared faster than LRT specimens, suggesting that traditional nasopharyngeal diagnostic techniques can miss cases of severe adenovirus infection. This suggests that LRT specimens are more reliable for diagnosing severe adenovirus infection, especially in patients with pneumonia that occurs several days after the infection onset when the frequency of virus detection in the URT has already decreased.

Several studies have shown that the mortality of severe adenovirus-associated ARDS can be as high as 26.7-80% in adults (10, 31). In our study, among the 14 patients with adenovirus-associated ARDS, there were only two deaths, and the mortality rate was 14.29%. Compared with previous studies, the mortality rate of adenovirus-associated ARDS in this study was relatively low, and this may have several explanations. First, rapid identification of adenovirus viral infection and early intervention are important to reduce the overall mortality rate. In this study, the time from onset to intubation was relatively short. In addition, the PO_2_/FiO_2_ ratio for most adenovirus-associated ARDS patients was >150 in this study, while most other studies showed that the PO_2_/FiO_2_ ratio in patients with severe pneumonia was <150 (6, 23, 31). Considering that the condition of severe adenovirus pneumonia patients was more advanced, the above results suggested that early intervention in adenovirus pneumonia-related ARDS helped to improve the patients' prognosis. Second, establishing organ support, such as application of early renal replacement treatment and ECMO are important. Adenovirus-associated ARDS completely resolved in three patients who were supported by ECMO in this study, suggesting that early application of ECMO improved the prognosis of patients with adenovirus-associated ARDS. Finally, timely initiation of antiviral therapy is very important to improve patient outcome. Currently, antiviral therapies for adenovirus infection remain controversial. No specific and effective antiviral drug is available for adenovirus infection (32). Some studies have shown that cidofovir antiviral therapy in severe adenovirus pneumonia improves the clinical prognosis (33). However, clinical application of cidofovir is limited due to its toxic side effects and low-quality evidence. Other case reports have also shown that ribavirin can be used to treat adenovirus infection (34, 35). Ganciclovir has been shown to be effective for treating adenovirus infection in animal experiments (36). In our study, 11 patients (78.57%) received antiviral therapy with ganciclovir and immunoglobulin therapy after confirming adenovirus infection, which might be the reason for the lower mortality.

This study prospectively observed the viral etiology of ventilated ARDS patients, especially for patients where adenovirus was associated with ARDS. We compared the adenovirus detection rate and adenovirus load in different respiratory tract specimens, which were also a highlight of the study. This study also has several limitations. It was a single-center study with a relatively small number of patients enrolled. In addition, this study used multiplex Q-PCR to detect multiple respiratory viruses at the same time without genotyping the adenovirus.

## Conclusions

Our findings indicated that adenovirus infection was an important cause of viral-related ARDS. The detection rate of adenovirus from the LRT was higher than that from the URT. Age, lower CD3+CD4+ T cells, and high serum creatinine may be were associated with adenovirus induce ARDS in adults required mechanical ventilation. Early identification and effective intervention to prevent disease progression are essential for reducing the mortality rate in these patients.

## Data Availability Statement

The original contributions presented in the study are included in the article/supplementary material, further inquiries can be directed to the corresponding author/s.

## Ethics Statement

The studies involving human participants were reviewed and approved by the Ethics Committee of the First Affiliated Hospital of Guangzhou Medical University (2019-19). The patients/participants provided their written informed consent to participate in this study. Written informed consent was obtained from the individual(s) for the publication of any potentially identifiable images or data included in this article.

## Author Contributions

XiL: study conception and design. ZW, RZ, DL, XuL, SC, YX, JZ, and ZZ: data collection. ZW, RZ, WH, XiL, and YL: analysis and interpretation. RZ and ZW: writing the manuscript. All authors have read and approved the final manuscript.

## Funding

This study was supported by National Natural Science Foundation of China (Nos. 82070084 to XiL and 81700080 to RZ) and National Science and Technology Major Project (No. 2017ZX10204401003).

## Conflict of Interest

The authors declare that the research was conducted in the absence of any commercial or financial relationships that could be construed as a potential conflict of interest.

## Publisher's Note

All claims expressed in this article are solely those of the authors and do not necessarily represent those of their affiliated organizations, or those of the publisher, the editors and the reviewers. Any product that may be evaluated in this article, or claim that may be made by its manufacturer, is not guaranteed or endorsed by the publisher.
